# Prognostic value of coronary risk factors, exercise capacity and single photon emission computed tomography in liver transplantation candidates: A 5-year follow-up study

**DOI:** 10.1007/s12350-020-02126-z

**Published:** 2020-05-11

**Authors:** William E. Moody, Benjamin Holloway, Parthiban Arumugam, Sharon Gill, Yasmin S. Wahid, Chris M. Boivin, Louise E. Thomson, Daniel S. Berman, Matthew J. Armstrong, James Ferguson, Richard P. Steeds

**Affiliations:** 1 Department of Nuclear Medicine, Centre for Clinical Cardiovascular Science, Nuffield House, Queen Elizabeth Hospital Birmingham, University Hospital Birmingham NHS Foundation Trust, Edgbaston, B15 2TH UK; 2 Department of Nuclear Medicine, Manchester Royal Infirmary, Manchester University NHS Foundation Trust, Oxford Road, Manchester, M13 9WL UK; 3 Department of Liver Medicine, Queen Elizabeth Hospital Birmingham, Edgbaston, B15 2TH UK; 4 Institute of Immunology and Immunotherapy, National Institute for Health Research (NIHR) Birmingham Liver Biomedical Research Centre (BRC), University of Birmingham, Birmingham, UK; 5 Departments of Imaging and Medicine, S. Mark Taper Foundation Imaging Center, Cedars-Sinai Medical Center, Los Angeles, USA

**Keywords:** SPECT, diagnostic and prognostic application, outcomes research, exercise testing, vasodilators

## Abstract

**Background:**

Although consensus-based guidelines support noninvasive stress testing prior to orthotopic liver transplantation (OLT), the optimal screening strategy for assessment of coronary artery disease in patients with end-stage liver disease (ESLD) is unclear. This study sought to determine the relative predictive value of coronary risk factors, functional capacity, and single photon emission computed tomography (SPECT) on major adverse cardiovascular events and all-cause mortality in liver transplantation candidates.

**Methods:**

Prior to listing for transplantation, 404 consecutive ESLD patients were referred to a University hospital for cardiovascular (CV) risk stratification. All subjects met at least one of the following criteria: inability to perform > 4 METs by history (62%), insulin-treated diabetes mellitus (53%), serum creatinine > 1.72 mg/dL (8%), history of MI, PCI or CABG (5%), stable angina (3%), cerebrovascular disease (1%), peripheral vascular disease (1%). Subjects underwent Technetium-99m SPECT with multislice coronary artery calcium scoring (CACS) using exercise treadmill or standard adenosine stress in those unable to achieve 85% maximal heart rate (Siemens Symbia T16). Abnormal perfusion was defined as a summed stress score (SSS) ≥ 4.

**Results:**

Of the 404 patients, 158 (age 59 ± 9 years; male 68%) subsequently underwent transplantation and were included in the primary analysis. Of those, 50 (32%) died after a mean duration follow-up of 5.4 years (maximal 10.9 years). Most deaths (78%) were attributed to noncardiovascular causes (malignancy, sepsis, renal failure). Of the 32 subjects with abnormal perfusion (20%), nine (6%) had a high-risk perfusion abnormality defined as a total perfusion defect size (PDS) ≥ 15% and/or an ischemic PDS ≥ 10%. Kaplan–Meier survival curves demonstrated abnormal perfusion was associated with increased CV mortality (generalized Wilcoxon, *P* = 0.014) but not all-cause death. Subjects with both abnormal perfusion and an inability to exercise > 4 METs had the lowest survival from all-cause death (*P* = 0.038). Abnormal perfusion was a strong independent predictor of CV death (adjusted HR 4.2; 95% CI 1.4 to 12.3; *P* = 0.019) and MACE (adjusted HR 7.7; 95% CI 1.4 to 42.4; *P* = 0.018) in a multivariate Cox regression model that included age, sex, diabetes, smoking and the ability to exercise > 4 METs. There was no association between CACS and the extent of perfusion abnormality, nor with outcomes.

**Conclusions:**

Most deaths following OLT are noncardiovascular. Nonetheless, abnormal perfusion is prevalent in this high-risk population and a stronger predictor of cardiovascular morbidity and mortality than functional status. A combined assessment of functional status and myocardial perfusion identifies those at highest risk of all-cause death. (Exercise Capacity and Single Photon Emission Computed Tomography in Liver Transplantation Candidates [ExSPECT]; ClinicalTrials.gov Identifier: NCT03864497).

**Electronic supplementary material:**

The online version of this article (10.1007/s12350-020-02126-z) contains supplementary material, which is available to authorized users.

## Introduction

There is ongoing debate over the optimal screening method for coronary artery disease (CAD) in patients with end-stage liver disease (ESLD) being considered for transplantation. Although screening is widely practiced, no current strategy has yet been shown to improve outcomes in these subjects. In 2012, the American Heart Association and American College of Cardiology (AHA/ACC) published guidelines suggesting that regardless of functional status, the presence of ≥ 3 cardiovascular (CV) risk factors (age ≥ 60 years, diabetes mellitus, smoking, hypertension, dyslipidemia, left ventricular hypertrophy) should prompt screening of asymptomatic candidates under consideration for orthotopic liver transplantation (OLT).^1^ This was a consensus-based recommendation (Class IIb, Level of Evidence C), which reflects the limited quality of data evaluating the efficacy of perioperative risk stratification in this population. Those retrospective observational studies that are available report low sensitivity and specificity for *both* dobutamine stress echocardiography (DSE)^2^-^5^ and single photon emission computed tomography (SPECT) imaging in OLT candidates.^6^,^7^ This may in part, relate to the high resting myocardial blood flows associated with cirrhotic liver disease, which may lend itself poorly to pharmacological vasodilator stress.^6^,^8^ Nonetheless, in 2013 the American Association for the Study of Liver Diseases and American Society of Transplantation guidelines proposed DSE as “an effective screening test” in OLT candidates.^9^,^10^ In reality, most liver transplant centers tailor their choice of noninvasive stress imaging according to local expertise. This practice is supported by the latest data suggesting SPECT has equivalent efficacy compared with DSE in the identification of clinically significant CAD,^11^ and in keeping with an ACC Appropriate Use Criteria Task Force Report, which rated both modalities “appropriate” for ESLD subjects with poor functional capacity (< 4 METs).^12^

The AHA/ACC recommendation to perform noninvasive screening in high-risk OLT candidates is supported by the finding that obstructive CAD on invasive angiography is more common in subjects referred with conventional risk factors.^13^ Despite this observation, traditional CV risk factors tend to perform poorly at predicting increased CV event rates in the ESLD population.^14^-^17^ In a retrospective analysis of 413 subjects undergoing liver transplant surgery, despite including *all* conventional CV factors in multivariate modeling, only a history of CAD or stroke were significant predictors of adverse cardiac outcomes at 30 days.^14^ In contrast, cardiopulmonary exercise testing (CPET) with measurement of estimated METs or maximum aerobic capacity, may offer more useful prognostic information in OLT candidates.^18^ Reduced aerobic capacity has been associated with worse outcomes following liver transplantation, although follow-up was limited to 90^19^ and 100 days.^20^

Controversy over whether SPECT imaging offers clinical utility in ESLD has been fueled by the conflicting reports on its accuracy to diagnose CAD in this setting.^6^,^7^,^11^,^21^ Moreover, there are no studies examining whether SPECT imaging provides any incremental prognostic value beyond functional testing in this high-risk cohort. We sought to determine, therefore, the relative importance of CV risk factors, functional capacity and SPECT perfusion as independent predictors of adverse outcome in OLT candidates.

## Methods

### Population

ExSPECT (Exercise Capacity and Single Photon Emission Computed Tomography in Liver Transplantation Candidates) was an observational cohort study, including consecutive ESLD patients referred for cardiac evaluation between September 2007 and June 2018, prior to consideration for liver transplantation. In accordance with local guidelines, subjects were referred for noninvasive CV risk assessment if they fulfilled any of the following criteria: inability to perform > 4 METs by history, insulin-treated diabetes mellitus, serum creatinine > 1.72 mg/dl, history of MI, PCI or CABG, stable angina, cerebrovascular disease, or peripheral vascular disease. The conduct and reporting of this study was in line with the principles of the Declaration of Helsinki and guided by the STROBE (Strengthening the Reporting of Observational Studies in Epidemiology) Statement.^22^ The study was approved by the University Hospital Birmingham NHS Divisional Clinical Quality Group (CARMS-14536) and was registered with ClinicalTrials.gov (NCT03864497).

### Clinical Data

The data that support the findings of this study are available from the corresponding author upon reasonable request. Demographic and anthropometric data were prospectively entered onto the patient’s electronic record. In addition, a standardized pre-scan clinical assessment was carried out including a detailed interview which elicited patient symptoms, CV risk factors, previous CV events, the cause for liver disease, the model for end-stage liver disease (MELD) score and medication history. Routine hematology and biochemistry indices at the time of the SPECT/CT imaging were also recorded. Diabetes mellitus (DM) was defined as a fasting glucose > 126 mg/dL, previous history of diabetes mellitus, or currently receiving hypoglycemic treatment. Hypertension was defined as an office blood pressure > 140/90 mmHg, or currently taking antihypertensive medication. Hypercholesterolemia was defined as a serum cholesterol of > 193 mg/dL, or currently taking lipid reduction therapy. History of CV disease included known coronary artery disease (myocardial infarction, previous percutaneous or surgical revascularization), heart failure, stroke, and peripheral vascular disease. Significant family history of CV disease was defined as a first degree relative with a history of myocardial infarction or ischemic stroke aged younger than 55 years in men and younger than 65 years in women.

### SPECT/CT

Patients were asked to discontinue β-blockers, rate-limiting calcium channel blockers, and caffeine products 24 hours before testing, and nitrate compounds were discontinued more than 6 hours before testing. All participants underwent 2-day stress-rest Technetium-99m SPECT imaging (Symbia T16, Siemens, Erlangen, Germany). Those patients capable of exercise underwent treadmill stress. Standard adenosine stress was performed (140 μg/kg/min for 6 minutes) in those subjects unable to exercise or unable to achieve 85% of age-predicted maximal heart rate. Hemodynamic response to adenosine was assessed by comparing hemodynamics at rest and after 3 to 4 minutes of adenosine infusion, immediately prior to radiotracer administration. An inadequate response was defined as a decrease in systolic blood pressure of < 10 mmHg or heart rate increase < 10 beats/min with adenosine infusion.^23^ CT-based attenuation correction was performed in all patients during reconstruction of the SPECT data. Multislice coronary artery calcium scoring (CACS) was also performed as routine in subjects without a history of myocardial infarction or coronary revascularization.

SPECT myocardial perfusion images were visually analyzed by two experienced observers (R.P.S. and B.H.) blinded to outcome variables (Hermes Medical Solutions, Quantitative Perfusion SPECT, Stockholm, Sweden). In addition to examination of raw images in cine mode, both nonattenuated and attenuated images were reviewed and a report was produced consistent with recommendations from the American Society of Nuclear Cardiology Imaging Guidelines for Nuclear Cardiology Procedures.^24^ Short-axis and vertical long-axis tomograms were divided into 17 segments for each study,^24^ and segmental tracer uptake was evaluated using a validated semi-quantitative 5-point scoring system (0, normal; 1, equivocal; 2, moderate; 3, severe reduction of radioisotope uptake; and 4, absence of detectable tracer uptake).^25^ The summed stress and rest scores were obtained by adding the scores of the 17 segments of the respective images. The sum of the differences between each of the 17 segments from these images was defined as the summed difference score, representing the amount of ischemia. These indices were converted to the percentage of total myocardium involved with stress, ischemic, or fixed defects by dividing the summed scores by 68 (the maximum potential score = 4 × 17), and multiplying by 100. The presence of abnormal perfusion was defined as a summed stress score (SSS) of 4 or greater.^26^ A stress-induced total perfusion defect size (PDS) > 15% or an ischemic PDS > 10% defined high risk for cardiac events.^27^ Cardiac volumes and left ventricular ejection fraction were calculated from the gated SPECT images (QGS 2012, Cedars-Sinai Medical Center, Los Angeles, CA, USA).

The CACS was calculated according to Agatston *et al.*^28^ by the same two independent observers in subjects without known coronary artery disease blinded to outcome data. Lesions were manually traced on CT images before semiautomatic quantification derived vessel-specific scores were summated to yield the total CACS (SyngoVia, Leonardo, Siemens Medical Solutions, Forchheim, Germany). Minimal, mild, moderate, and severe coronary calcification were defined as Agatston scores of 0 to 10 U, 11 to 100 U, 101 to 400 U and > 400 U, respectively.^27^,^29^

### Exercise Stress Testing

Exercise treadmill testing (ETT) was carried out according to standard protocols (Bruce and modified Bruce) aiming to achieve > 85% of age-predicted maximal heart rate with a goal test duration of between 8 and 12 minutes.^30^ During exercise, data on symptoms, rhythm, heart rate, blood pressure (by indirect arm-cuff sphygmomanometry), and estimated workload in metabolic equivalents (METs) were recorded and entered prospectively into the patient electronic record. A positive test was defined as one showing 1mm of ST segment elevation or depression (of any morphology and in any lead except aVR) 80 msec after the end of the QRS complex any time after exercise was begun. A negative test was one without this degree of ST segment change and in which the patient achieved 85% of the maximum predicted heart rate for his age. All other tests were considered indeterminate.^31^ To improve the estimated accuracy of exercise capacity, patients were explicitly told not to lean on handrails during exercise. Estimated functional capacity in METs was estimated from the total time completed in the final stage^32^; a MET is a measure of oxygen consumption defined as 1 kcal/kg/hour (equivalent to 3.5 ml of Oxygen/kg/min), which represents basal, rest metabolic needs. The Bruce protocol formula was used for estimating maximal oxygen uptake (VO2 max).^33^

### Coronary Angiography

Those patients with a high-risk perfusion abnormality on SPECT MPI were considered for invasive coronary angiography. The decision to proceed to angiography +/− invasive revascularization was made by the individual treating physician after each case was discussed in a dedicated multidisciplinary team meeting.

### End Points

The primary outcome measure was cardiovascular death. Major adverse cardiovascular events (MACE) and all-cause mortality were examined separately as secondary outcomes. Major adverse cardiovascular events were defined as a composite endpoint of cardiac death, nonfatal myocardial infarction (MI), malignant arrhythmia, ischemic stroke, or hospitalization for congestive heart failure. Myocardial infarction was defined as a clinical (or pathologic) event caused by myocardial ischemia where there is evidence of myocardial injury or necrosis as defined by a rise and/or fall of cardiac biomarkers in the presence of typical symptoms or electrocardiographic changes, or imaging evidence of new loss of viable myocardium or new regional wall motion abnormality.^34^ For the primary analysis, patients who did not undergo liver transplant surgery during the study period were identified and excluded from follow-up because of the strong mortality benefit associated with OLT in cirrhotic liver disease (see Data Supplement for outcome analysis including all consecutive subjects). Similarly, patients who had revascularization procedures within 90 days of an abnormal baseline SPECT imaging study were excluded to remove outcomes driven temporally by this result.^35^

Every patient in the NHS has a unique identifier which enables outcomes to be tracked using the Hospital Episodes Statistics (HES) Database, an administrative data warehouse containing admissions to all National Health Service hospitals in England.^36^ It contains detailed records relating to individual patient treatments, with data extraction facilitated using codes on procedural classifications (Office of Population Censuses and Surveys Classification of Interventions and Procedures, 4th revision (OPCS-4)) and medical classifications (World Health Organization International Classification of Disease, 10th revision (ICD-10)).^37^,^38^ With regard to outcome analysis, HES data alone have the limitation of only capturing deaths occurring in a hospital setting. To obtain the complete mortality list, the study cohort was also cross-referenced with mortality data from the Office for National Statistics (ONS), which collects information on all registered deaths in the UK.

An endpoint committee consisting of a cardiologist (W.E.M) and a hepatologist (J.F) adjudicated all outcomes by consensus. Outcomes were further verified by cross-referencing with individual hospital case notes held electronically. The endpoint committee was blinded to all clinical and imaging results.

### Statistics

Statistical analyses were performed with SPSS version 25 (IBM, Armonk, New York, US). Data are expressed as mean ± SD, median (interquartile range), or frequency (%), unless otherwise stated. The normality of distribution for continuous variables was determined using normality plots and the Kolmogorov-Smirnov test. Baseline characteristics of the population were examined by SPECT results. The Mann-Whitney U test was used to compare continuous nonparametric data. The Kruskal-Wallis analysis of variance was used to identify significant differences in central tendencies of continuously scaled variables between groups. Contingency table analysis was performed using Chi-square or Fisher’s exact tests where appropriate.

Annualized mortality rates are expressed as the number of patients having cardiovascular or all-cause death as a proportion of the number of patients at risk divided by the number of patient-years follow-up. Kaplan–Meier analysis of outcomes was based on discrete SPECT and exercise capacity categories. The date of the imaging test was used as time zero. Two-sided generalized Wilcoxon tests were used to determine significance. Multivariate Cox proportional hazards models were used to identify the association between time-to-event and baseline clinical characteristics, estimated METs, and SPECT results. The change in the global Chi-square statistic was calculated to determine the incremental prognostic value of clinical, exercise and SPECT data; the Chi-square of the model was calculated from the log likelihood ratio. A *P* value <0.05 was considered statistically significant for all analyses.

## Results

**Figure 1:**
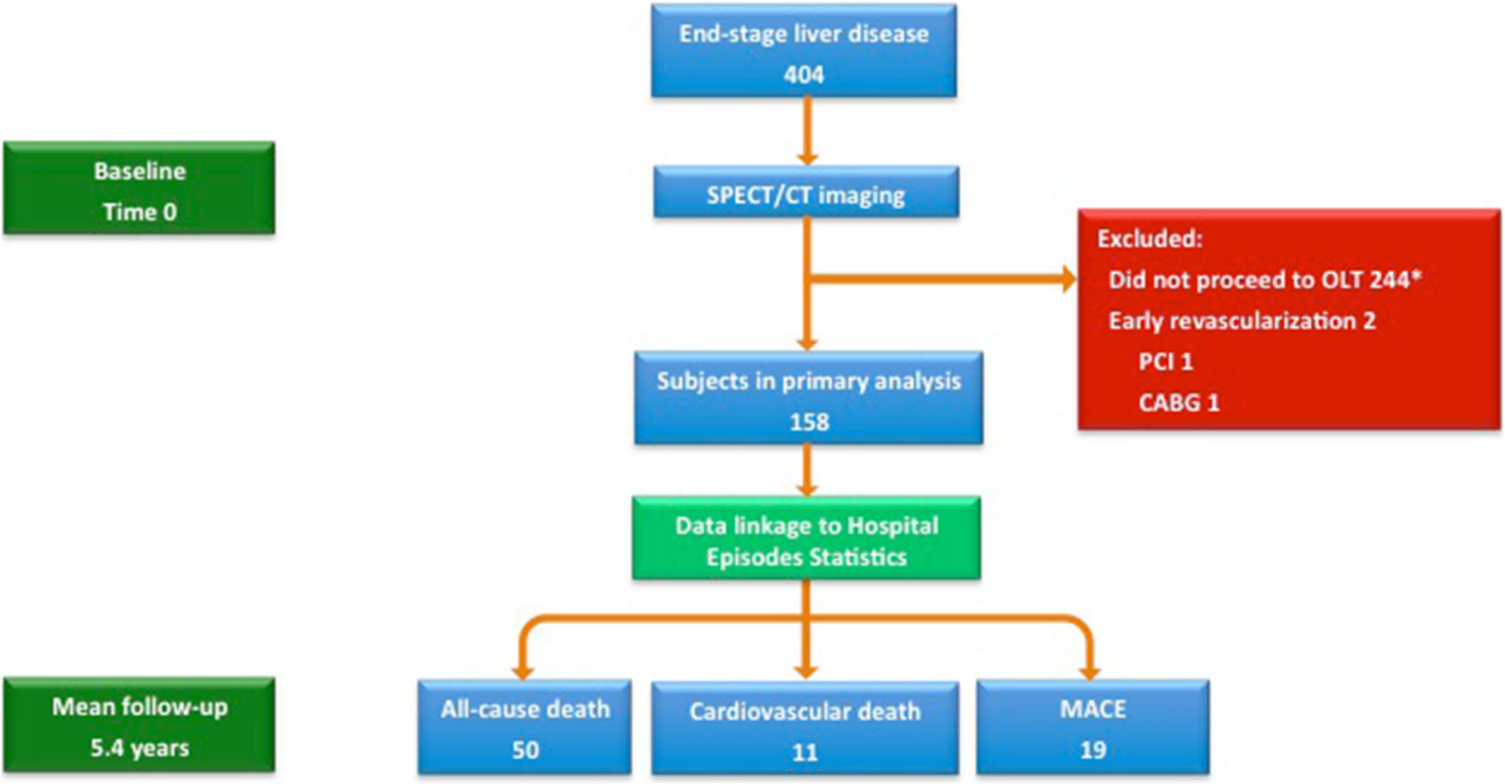
Study consort diagram. Abbreviations: *OLT*, orthotopic liver transplantation; *PCI*, percutaneous coronary intervention; *CABG*, coronary artery bypass graft surgery

Of the 404 patients with ESLD undergoing SPECT imaging between September 2007 and June 2018, 160 subjects subsequently underwent OLT. Reasons for patients not proceeding to OLT included compensated cirrhosis, extra-hepatic malignancy, hepatopulmonary syndrome, hepatorenal syndrome, severe obesity, malnutrition, active substance abuse, and psychosocial issues. Two patients without a prior diagnosis of coronary atheroma were excluded after undergoing early revascularization driven by the SPECT imaging demonstrating an ischemic PDS ≥ 10%. This resulted in 158 subjects available for inclusion in the present analysis (Figure 1). The demographics and outcomes for *all* consecutive patients (*n* = 404) are provided in the Data Supplement.

The baseline demographics and clinical characteristics of the study cohort are summarized in Table 1. Mean age was 57 years, 70% were male, 50% were diabetic, and 11% were hypertensive. Almost all patients were asymptomatic (97%). Table 2 summarizes the results of the functional assessment and imaging characteristics for all included subjects. The median time between SPECT imaging and liver transplant was 4 months (IQR 2.1 to 9.0)

**Table 1:** Baseline demographics and clinical characteristics for study cohort

Variable	N = 158
Age (years)	56.6 ± 8.9
Male sex	110 (70%)
Ethnicity	
White	135 (85%)
Asian	20 (13%)
Afro-Caribbean	1 (1%)
Other	2 (1%)
Body mass index (kg/m^2^)	29.9 ± 6.1
Etiology of end-stage liver disease	
Alcohol	36 (23%)
Hepatitis B	1 (1%)
Hepatitis C	21 (17%)
Nonalcoholic steatohepatitis	29 (19%)
Primary biliary cirrhosis	10 (6%)
Cryptogenic	2 (1%)
Autoimmune	2 (1%)
Alpha-1-antitrypsin deficiency	2 (1%)
Other	35 (29%)
MELD score	16 ± 6
Cardiac risk factors	
Diabetes^a^	79 (50%)
Hypertension	17 (11%)
Hypercholesterolemia	42 (27%)
Current smoker	28 (18%)
Family history of CAD	2 (1%)
Number of cardiac risk factors	1.7 ± 1.0
Symptomatic chest pain	2 (1%)
Typical angina/atypical or noncardiac	1 (1%)/1 (1%)
History of revascularization (PCI or CABG)	5 (3%)
History of myocardial infarction	5 (3%)
Medications	
Aspirin	17 (11%)
Beta-blocker	77 (49%)
ACE inhibitor/angiotensin receptor blocker	15 (9%)
Calcium channel blocker	2 (1%)
Loop diuretic	40 (25%)
Mineralocorticoid receptor antagonist	50 (32%)
Statin	35 (22%)
Insulin	77 (49%)
Hemoglobin (g/L)	117 (105−129)
Total cholesterol (mg/dL)	157 ± 54
INR	1.36 ± 0.33
Creatinine (mg/dL)	0.90 (0.75−1.15)

Data are number (%), mean ± SD or median (IQR)

^a^On Insulin or oral hypoglycemic therapy

*ACE*, angiotensin converting enzyme; *CABG*, coronary artery bypass graft surgery; *CAD*, coronary artery disease; *IQR*, interquartile range; *PCI*, percutaneous coronary intervention

**Table 2:** Functional assessment and imaging characteristics for study cohort

Variable	N = 158
Exercise treadmill stress	77 (49%)
METs achieved^a^	6.3 ± 2.4
Peak VO2 achieved (ml/kg/min)^a^	22.2 ± 8.4
Positive/indeterminate/negative^a^	2 (3%) / 13 (17%) / 61 (80%)
LV ejection fraction, %	60.9 ± 9.7
Abnormal SPECT^b^	32 (20%)
Summed stress score ≥ 9	9 (6%)
Total PDS ≥ 15%	9 (6%)
Ischemic PDS ≥ 10%	6 (4%)
CACS, Agatston units (IQR)^c^	11 (0–119)

Data are number (%), mean ± SD or median (IQR)

^a^In the 77 patients that were capable of exercise treadmill exercise stress

^b^Defined as summed stress score of 4 or more

^c^In the 84 patients for whom data were available

CACS, coronary artery calcium score; LV, left ventricular; METs, metabolic equivalents; PDS, perfusion defect size; SPECT, single photon emission computed tomography; VO2, oxygen uptake

### SPECT/CT

Abnormal perfusion was identified in 32 of the 158 patients (20%). Of those, 9 subjects (6%) had a high-risk perfusion abnormality, which was associated with a lower left ventricular ejection fraction on gated analysis (Table 3). There was no difference in the mean number of cardiac risk factors between subjects with a normal SPECT result and those with abnormal perfusion or a high-risk perfusion abnormality. There was, however, a graded association between the size of perfusion defect and the proportion of subjects with hypercholesterolemia. Subjects with abnormal perfusion on SPECT showed a trend toward higher CAC scores but this result did not reach significance (*P* = 0.087, Table 3).

**Table 3:** Baseline demographics, clinical characteristics, and stress test differences by SPECT results (N = 158)

Variable	Normal perfusion (N = 126)	Abnormal perfusion (N = 32)	*P* value^a^	Total PDS <15% (N = 23)	Total PDS ≥15% (N = 9)	*P* value^b^
Age	56.5 ± 7.9	56.9 ± 8.3	0.78	57.5 ± 9.1	55.0 ± 6.0	0.16
Male	84 (67%)	26 (81%)	0.10	19 (83%)	7 (78%)	0.43
Cardiac risk factors						
Diabetes	63 (50%)	16 (50%)	0.51	13 (57%)	3 (33%)	0.19
Hypertension	15 (12%)	2 (6%)	0.33	2 (9%)	0 (0%)	0.46
Hypercholesterolemia	27 (21%)	15 (47%)	0.26	10 (44%)	5 (56%)	0.02
Smoking history	27 (21%)	7 (22%)	0.75	5 (22%)	2 (22%)	0.92
Able to perform exercise stress	61 (48%)	16 (50%)	0.81	11 (48%)	5 (56%)	0.89
METs^c^	6.6 ± 2.5	5.5 ± 1.6	0.15	5.1 ± 1.1	7.4 ± 2.8	0.12
LV ejection fraction, %	62.2 ± 9.7	55.5 ± 8.2	0.01	56.6 ± 7.1	54.4 ± 9.8	0.02
CACS, Agatston units (IQR)^d^	7 (0–113)	38 (11–260)	0.09	48 (11–419)	11 (1–36)	0.52

Data are N (%), mean ± SD or median (interquartile range)

*CACS*, coronary artery calcium score; *METs*, metabolic equivalents; *PDS*, perfusion defect size; *LV*, left ventricular

^a^Normal perfusion versus abnormal perfusion

^b^Normal SPECT versus total PDS <15% versus total PDS ≥15%. The Kruskal-Wallis analysis of variance was used to identify significant differences in central tendencies of continuously scaled variables between groups. Contingency table analysis was performed using Chi-square or Fisher’s exact tests where appropriate

^c^In the 77 subjects capable of treadmill exercise

^d^Coronary artery calcium scoring was performed in 84 patients without a known history of myocardial infarction or revascularization and with resting heart rates < 80 bpm. The numbers of patients per subgroup were as follows: normal perfusion (*n* = 60), abnormal perfusion (*n* = 24); total PDS <15% (*n* = 20); total PDS ≥15% (*n* = 4)

**Figure 2:**
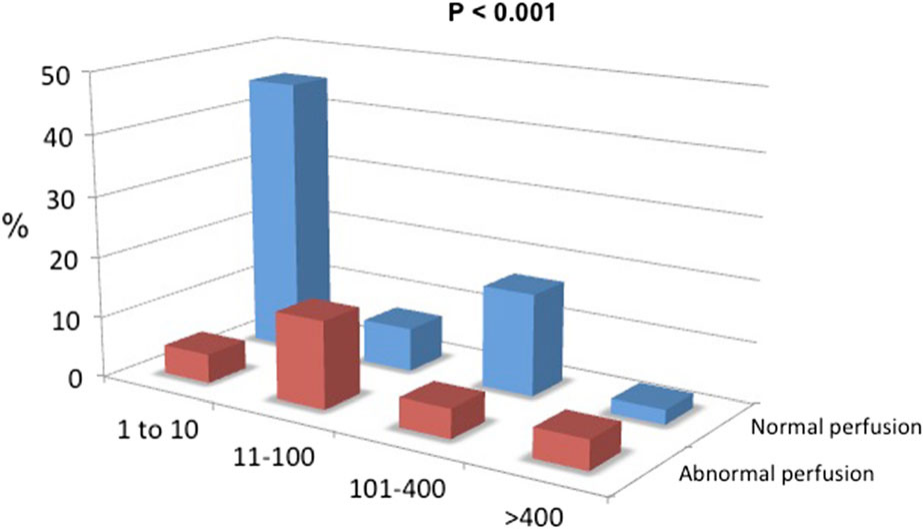
Relation between CACS severity and stress SPECT results (*n* = 84). The percentage of subjects with a normal SPECT result was highest in those with minimal CACS (*P* < 0.001). A 4 × 2 contingency analysis was performed using a two-tailed Fisher’s exact test to determine significance

The relationship between CACS and SPECT results is displayed in Figure 2. Most patients had zero, minimal or mild CAC (71%). The majority of those with minimal CACS (90%) had normal perfusion, although 4 subjects (10%) with minimal CACS did demonstrate a high-risk SPECT profile based on a stress-induced total PDS ≥ 15%. Those subjects with at least moderate CAC (29%) were more likely to have diabetes (Chi-square = 4.2, P = 0.04) although there was no association between increasing CACS and any other traditional risk factor.

### Exercise

There was no significant association between the ability to perform treadmill exercise and the likelihood of having a perfusion defect or the CACS severity. In the 77 patients (49%) who underwent exercise stress, there was no association between the number of METS achieved and the likelihood of an abnormal perfusion result (Table 3). There was, however, a weak correlation between increasing CACS and reduced METS (*ρ* = − 0.45, *P* = 0.046).

### Coronary Angiography

Of the 9 subjects with a high-risk perfusion abnormality by SPECT (total PDS ≥ 15%), 6 patients had an ischemic PDS ≥ 10%. Of those, 3 subjects proceeded to invasive coronary angiography. Subsequently, 2 patients were diagnosed with nonobstructive coronary disease and 1 patient with obstructive coronary disease.

### Outcomes

**Figure 3:**
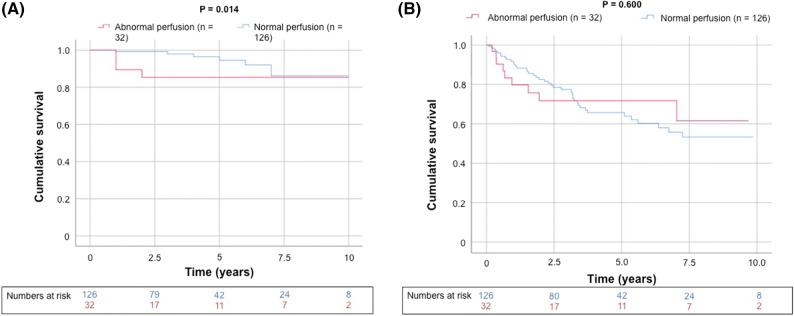
Kaplan–Meier curve for unadjusted cumulative survival from (**A**) cardiovascular death and (**B**) all-cause death according to perfusion dichotomized by a summed stress score ≥ 4. Two-sided generalized Wilcoxon tests were used to determine significance

**Figure 4:**
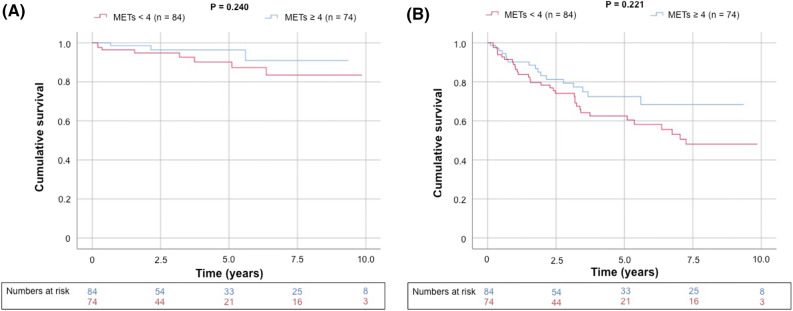
Kaplan–Meier curve for unadjusted cumulative survival from (**A**) cardiovascular death and (**B**) all-cause death according to functional capacity dichotomized by estimated METs ≤ 4. Two-sided generalized Wilcoxon tests were used to determine significance

**Figure 5:**
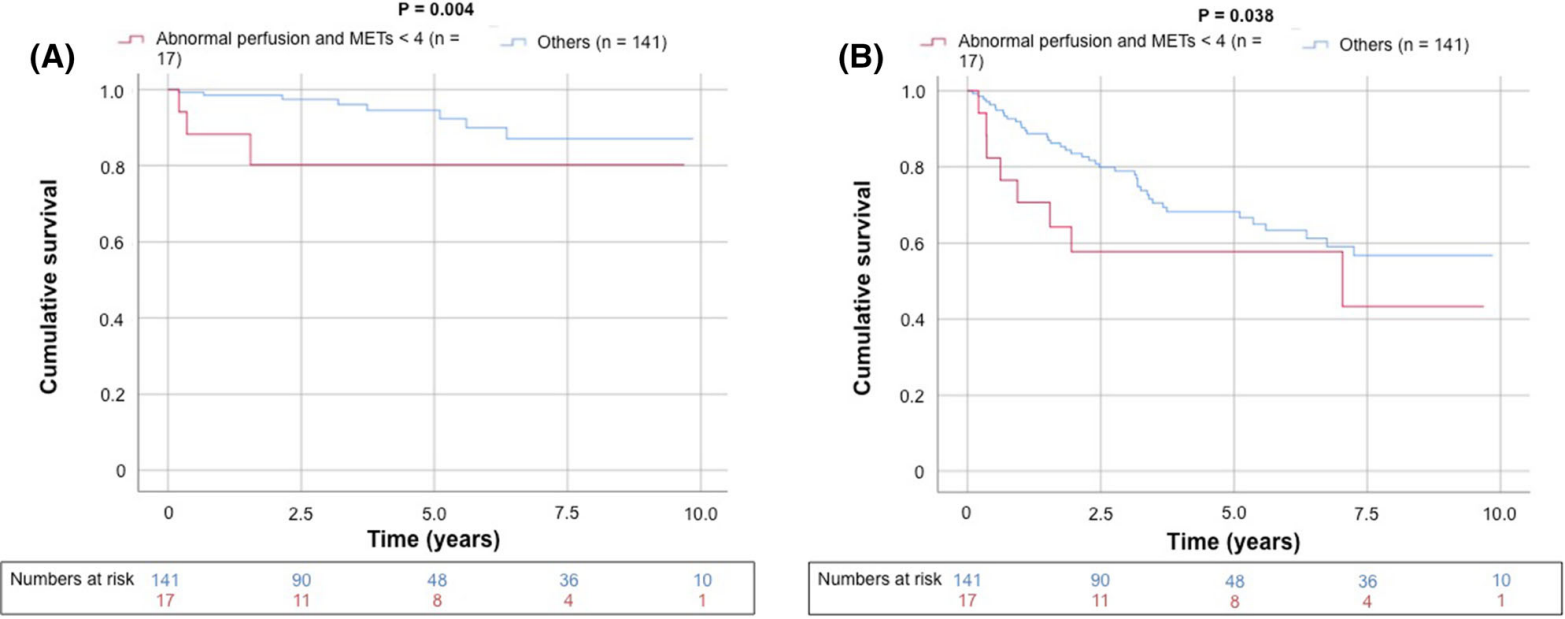
Kaplan–Meier curve for unadjusted cumulative survival from (**A**) cardiovascular death and (**B**) all-cause death according to integrated results of SPECT and exercise capacity. Two-sided generalized Wilcoxon tests were used to determine significance

**Figure 6:**
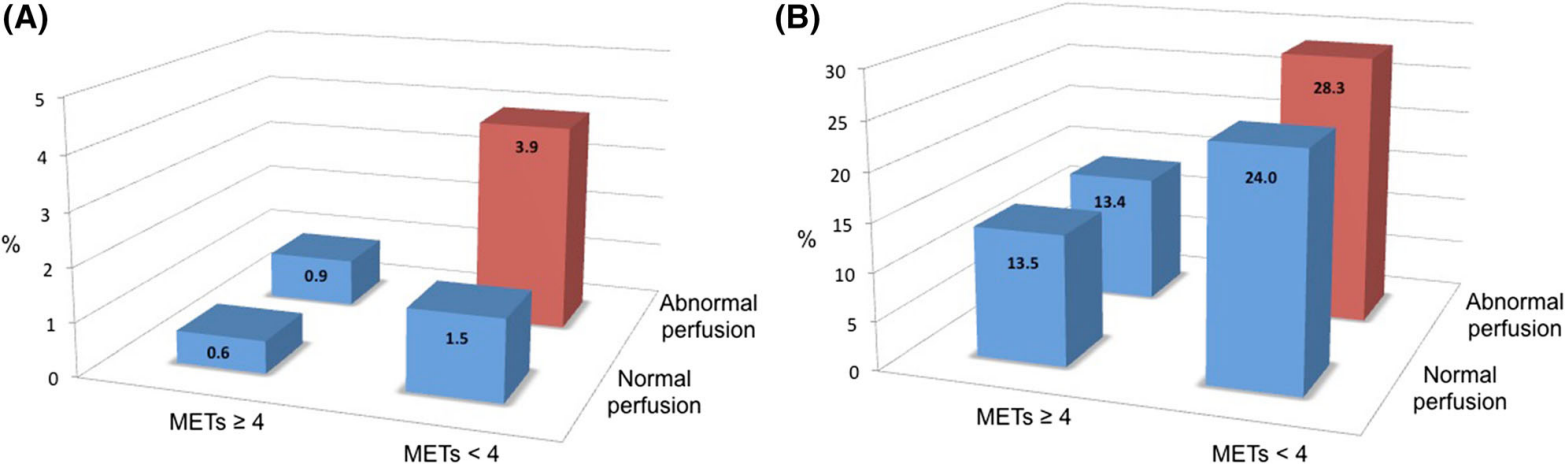
Annualized event rates for (**A**) cardiovascular death and (**B**) all-cause death according to integrated results of SPECT and exercise capacity. (**A**) For each subgroup (number of CV deaths/number of patients): METs ≥ 4, normal perfusion (1/59); METs <4, normal perfusion (4/67); METs ≥ 4, abnormal perfusion (2/15); METS <4, abnormal perfusion (4/17). (**B**) For each subgroup (number of all-cause deaths / number of patients): METs ≥ 4, normal perfusion (15/60); METs <4, normal perfusion (26/66); METs ≥ 4, abnormal perfusion (2/15); METS <4, abnormal perfusion (7/17)

**Figure 7:**
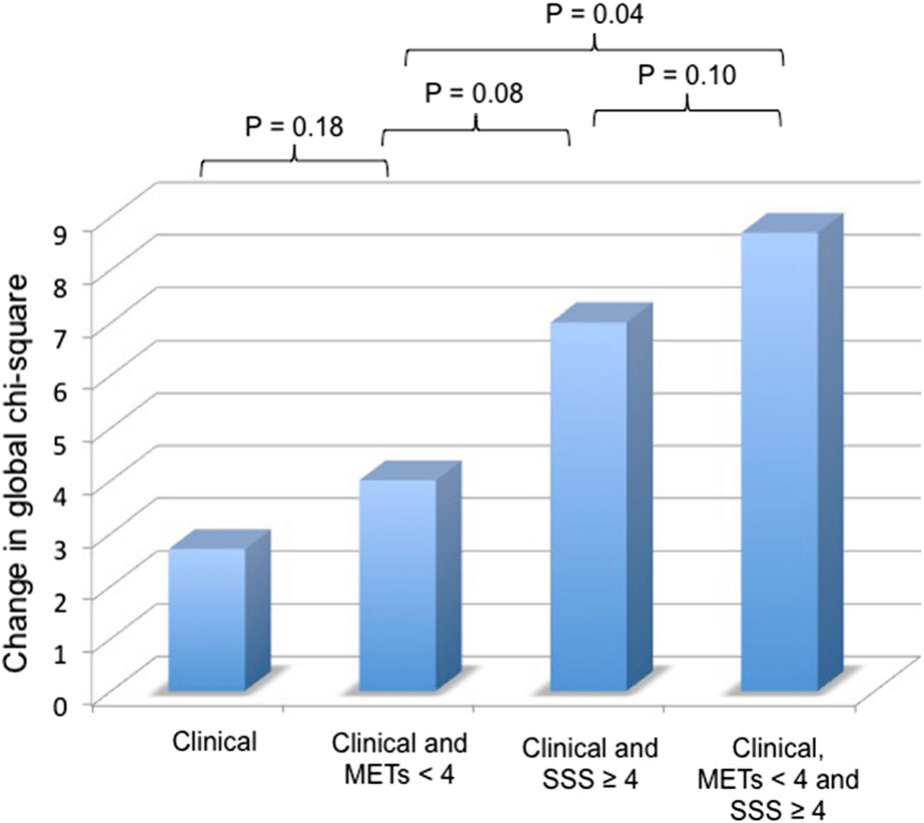
Incremental predictive value of exercise capacity and stress SPECT results over clinical information to predict cardiovascular death. The clinical data entered into the global Chi-square analysis model included age, sex, diabetes, smoking history. Abnormality on SPECT (defined as SSS ≥ 4) and exercise capacity (METs < 4) were entered as binary variables

There were a total of 50 deaths (32%) after a mean duration of 5.4 years (maximal follow-up 10.9 years), and most (78%) were attributed to noncardiovascular causes (Table 4). Kaplan–Meier survival curves demonstrate abnormal perfusion was associated with increased CV mortality (generalized Wilcoxon, *P* = 0.014) but not all-cause death (Figure 3). Although there was a trend toward increased CV mortality in those patients with impaired functional status (Figure 4), this result did not reach statistical significance (generalized Wilcoxon, *P* = 0.24). Similarly, on Kaplan–Meier survival analysis, for patients that underwent adenosine stress (i.e., those patients unable to perform treadmill exercise) there was a trend toward increased CV and all-cause mortality but this association did not reach significance (*P* = 0.398 and *P* = 0.791, respectively, Figure 4). There was, however, incremental predictive value when integrating the results of functional testing and SPECT imaging (Figures 5, 6, 7); subjects with *both* abnormal perfusion and an inability to exercise > 4 METs had elevated cardiovascular and all-cause mortality (generalized Wilcoxon, *P* = 0.004 and *P* = 0.038, respectively).

**Table 4:** Follow-up (N = 158)

Mortality, all-cause	50 (32%)
Cardiovascular	11
Infection	10
Bleeding	2
Malignancy	17
Multi-organ/renal failure	3
Recurrent liver disease	6
Other	1

Major adverse cardiac events	19 (12%)
Cardiovascular death	11
Fatal myocardial infarction	4
Sudden cardiac death	4
Ischemic stroke	3
Nonfatal myocardial infarction	1
Revascularization^a^	2
Aborted sudden cardiac death	2
Nonfatal ischemic stroke	2
Congestive heart failure	1

Values are N (%) or N

^a^Late revascularization > 90 days after the SPECT/CT imaging

Abnormal perfusion was an independent predictor of CV death albeit with wide confidence intervals (Table 5; adjusted HR 4.2; 95% CI 1.4 to 27.3; *P*=0.019) and MACE (adjusted HR 7.7; 95% CI 1.4 to 42.4; *P* = 0.018) in a multivariate Cox regression model that included age, sex, diabetes, smoking and the ability to exercise > 4 METs. There was no significant association between CACS and adverse outcomes. One patient with a minimal CACS suffered a sudden cardiac death.

**Table 5:** Multivariate predictors of 5-year cardiovascular mortality

Variable	Cardiovascular death	
	HR (95% CI)	*P* value
Age	0.95 (0.88–1.04)	0.272
Gender (female)	2.07 (0.56–7.64)	0.276
Diabetes	1.39 (0.87–7.59)	0.088
Current smoker	5.23 (1.07–25.46)	0.041
METs < 4	2.73 (0.60–12.54)	0.196
Abnormal perfusion^a^	4.18 (1.43–12.27)	0.019

All variables listed were simultaneously entered into a multivariate Cox regression model

*METs*, metabolic equivalents

^a^Defined as summed stress score ≥4

In subjects with abnormal perfusion by SPECT (SSS ≥ 4), the annualized cardiovascular death rate was 2.6% versus 1.0% in those with normal perfusion (*P* < 0.001). Subjects with abnormal perfusion *and* who were unable to exercise ≥ 4 METs experienced the highest annualized cardiovascular and all-cause mortality rates (3.9% and 28.3%, respectively; Figure 6). Only 7 out of the 126 subjects (5.5%) with normal SPECT perfusion suffered a CV death after a mean follow-up of 5.4 years; four of these subjects underwent adenosine stress and 3 had exercise stress testing.

Figure 7 depicts the incremental value of stress SPECT results over clinical data to predict cardiovascular death by global Chi-square analysis. There was a significant improvement in risk prediction with the addition of abnormal perfusion on SPECT to clinical information and exercise capacity (Chi-square change = 4.7, *P* = 0.04).

## Discussion

In the longest follow-up survival study of OLT candidates undergoing noninvasive stress imaging to date, we have shown that abnormal perfusion is common in this high-risk population (20%) and is a stronger predictor of cardiovascular morbidity and mortality than functional status. The addition of abnormal perfusion to coronary risk factors and exercise capacity provides incremental prognostic utility for the prediction of CV death. Nonetheless, most subjects die from non-CV causes and a combined assessment of functional status and myocardial perfusion identifies those at highest risk of all-cause mortality. Cardiorespiratory fitness (as defined by estimated METs) *and* abnormal perfusion (as determined by SPECT); therefore, *both* appear to be important predictors of adverse long-term outcomes post-liver transplantation.

There are limited quality studies guiding clinicians on risk stratification in the perioperative period (at the time of transplantation and in the postoperative period out to 6 months) but even less data addressing risk beyond 6 months, when the patient is thought to be more commonly on a reduced immunosuppression regime. To date, almost all follow-up studies involving noninvasive stress testing for risk stratification prior to OLT have focused on perioperative outcomes.^5^,^11^,^14^,^19^,^20^,^39^ One of the major advantages of the current study is its ability to inform clinicians on intermediate and long-term outcomes. This is particularly relevant because liver grafts have become an increasingly scarce commodity; clinicians, therefore, have an important duty to ensure that graft survival is not limited by premature CV death. Based on the current study, the incorporation of combined functional and perfusion data into pre-operative risk stratification should help facilitate appropriate resource allocation, which in turn may lead to better overall long-term outcomes for patients with ESLD.

In our institution, SPECT imaging is reserved for high-risk candidates; only 49% of subjects were capable of treadmill exercise stress and 50% were diabetic. Using this pre-op risk stratification strategy, the 5-year survival for this specific cohort was 68%. Taking into account the high-risk baseline profile of this selected population, this figure compares favorably with the unadjusted 5-year survival rate for adult liver transplant recipients (72%) reported by the U.S. Organ Procurement and Transplantation Network.^40^ In keeping with a study from Zoghbi *et al.*,^41^ which included 82 patients undergoing SPECT, most of our patients suffered a non-CV death, although the frequency of abnormal perfusion was notably higher in our cohort (20% vs. 9%). This finding might be explained by the higher proportion of males (70% vs. 63%) and active smokers (18% vs. 12%), as well as a higher prevalence of diabetes mellitus (50% vs. 16%).

A small proportion of subjects with normal SPECT perfusion (5.5%) suffered a CV death after an extended period of follow-up (mean 5.4 years), although only one occurred within the first 6 months. It is well recognized that the highest risk period is often at the time of donor liver reperfusion when unstable arrhythmia can be induced.^5^,^17^,^42^ It is conceivable that perioperative CV events might occur in the absence of ischemia in patients with lactatemia, acidosis, hypoxia, hypotension, or electrolyte disturbances, which may predispose to acquired electrocardiogram abnormalities such as a prolonged QT interval. Despite prior concerns about the sensitivity of vasodilator stress SPECT testing in patients with cirrhotic liver disease and high resting myocardial blood flows,^6^,^8^ the current data suggest it has the ability to detect prognostically important CV disease in the short- and intermediate-term.

There was incremental prognostic utility from adding SPECT perfusion data to traditional coronary risk factors and these findings contrast with a recent report suggesting a simple assessment of traditional CV risk factors was equivalent to SPECT imaging in its diagnostic ability to predict significant coronary artery disease.^7^ In our cohort, smoking was the only conventional risk factor independently associated with reduced CV survival. Roughly one half of this cohort constituted asymptomatic diabetic patients but in contrast to abnormal perfusion, the presence of diabetes was not an independent predictor of premature CV death. This finding needs to be taken into consideration with the results of the DIAD study,^43^ which showed that screening asymptomatic diabetic with SPECT MPI did not confer an improvement in CV outcomes over a similar follow-up period (4.8 years).

In the general population, there is a well-recognized inverse association between functional status and mortality, independent of age^44^, sex^45^,^46^, and ethnicity^47^. In a recent cohort study including 122,007 patients undergoing treadmill testing, the increase in all-cause mortality associated with reduced cardiorespiratory fitness (as defined by peak estimated metabolic equivalents (METs)) was comparable to or greater than for traditional CV risk factors.^48^ The current data support that functional status has an equally important prognostic role in patients with ESLD.

In a retrospective study of OLT candidates (*n* = 772) referred for screening SPECT at a single institution, only 8% of studies were deemed abnormal.^49^ A total of 26 patients with positive SPECT and angiographic evidence of CAD were denied transplantation; however, CAD was the only factor for denial in only 7 patients. The analysis demonstrated that the screening stress MPI results were not associated with liver transplantation eligibility and suggested that because of the large number of competing factors considered prior to transplantation and the low prevalence of abnormal stress MPI results, screening for CAD should be reserved for patients deemed otherwise acceptable for transplantation. The higher prevalence of abnormal SPECT perfusion in the current study suggests that reserving screening to asymptomatic patients who are at elevated baseline risk (unable to perform > 4 METs, presence of diabetes, smoking, renal dysfunction) is an acceptable strategy and might increase the influence of stress MPI findings.

Identifying advanced CAD in the form of detectable CAC may offer an alternative strategy to recognize vulnerable patients. Severe coronary artery calcification (CACS > 400 U) is predictive for revascularization,^50^ and of 30-day post-LT cardiovascular complications,^39^ although data supporting an increased risk in OLT patients after the initial postoperative period are lacking. Of concern, the current study demonstrates that a minimal CACS in ESLD patients does not exclude the possibility of finding abnormal perfusion on SPECT. Furthermore, we found no association between CACS and outcomes, although this study was not designed to address this issue and was likely underpowered. The presence of coronary atheroma *per se* is unlikely to be the sole factor for excluding a patient from proceeding to OLT. Increased CACS does not form an indication for revascularization at any level and at present, a decision to deny a patient transplantation driven by increased CAC seems incorrect.

Although 10% of patients with minimal coronary calcification had an abnormal SPECT result, only one such patient suffered a major adverse cardiovascular event (a sudden cardiac death) and there were no adverse events in any patient with a CACS of zero after a mean follow-up of 5 years. These findings support the pragmatic approach recently proposed in this journal,^51^,^52^ where coronary computed tomography angiography ± CACS can be used to exclude coronary disease with the added advantage of having a long warranty.

There are limitations to our study. These data are from consecutive patients recruited from a single center. The majority of patients were white males, which limits the generalizability of the results. Given that roughly half the cohort had diabetes, statin usage on study entry was relatively low (22%) and although baseline medications were recorded, we did not capture information on changes in medical therapy over the study period, which is a factor that may have influenced outcomes. A recent observational report suggested that so long as patients were appropriately revascularized, the severity or extent of CAD did not influence post-transplant survival, although follow-up was limited to only 90 days.^53^ We are unable to provide information on the influence of revascularization on OLT candidates because of the low numbers of subjects who underwent invasive coronary angiography. Our practice likely reflects the conclusions drawn from general population studies; randomized controlled trials have shown no benefit in prophylactic revascularization compared with medical therapy in stable patients undergoing surgery (even major vascular surgery) despite the presence of ischemia.^54^,^55^

## Conclusion

These data suggest that MPI SPECT provides incremental and independent prognostic value in OLT candidates. Based on the presence of risk factors, it appears reasonable to continue to risk stratify asymptomatic candidates with a noninvasive assessment that combines exercise testing and MPI SPECT imaging. More prospective evidence is required, however, ideally from randomized clinical trials, to guide the indications, timing, and outcomes of revascularization therapy in patients with ESLD.

## New Knowledge Gained

Abnormal perfusion as defined by MPI SPECT, is prevalent in the liver transplant population and an independent predictor of cardiovascular death. MPI SPECT provides complementary prognostic value to functional testing—a combined assessment of cardiorespiratory fitness and myocardial perfusion identifies those subjects at highest risk of cardiovascular and all-cause death.

## Electronic supplementary material

Below is the link to the electronic supplementary material.

Supplementary material 1 (DOCX 295 kb)

Supplementary material 2 (PPTX 531 kb)

## Abbreviations

CAD: Coronary artery disease
CACS: Coronary artery calcium score
CV: Cardiovascular
ESLD: End-stage liver disease
METs: Metabolic equivalents
OLT: Orthotopic liver transplantation
PDS: Perfusion defect size
SPECT: Single photon emission computed tomography
SSS: Summed stress score
