# Supplementary material for: Prognostic value of coronary risk factors, exercise capacity and single photon emission computed tomography in liver transplantation candidates: A 5-year follow-up study
Source: J Nucl Cardiol. 2020 May 11;28(6):2876–91. doi: 10.1007/s12350-020-02126-z (PMC8709822; doi:10.1007/s12350-020-02126-z)
Supplement: Supplementary file 1 — Supplementary material 1 (DOCX 295 kb) [file 12350_2020_2126_MOESM1_ESM.docx]

**Data Supplement**

**Table S1. Baseline demographics and clinical characteristics for all consecutive patients**

| **Variable** | **All subjects**  **N = 404** | **Liver transplant**  **N = 158** | **No liver transplant***  **N = 246** | **P value** |
| --- | --- | --- | --- | --- |
| Age (yrs.) | 58.8 ± 7.7 | 56.6 ± 8.9 | 60.2 ± 7.3 | 0.0001 |
| Male sex | 276 (68%) | 110 (70%) | 166 (67%) | 0.57 |
| Ethnicity |  |  |  |  |
| White | 355 (88%) | 135 (85%) | 220 (89%) | 0.54 |
| Asian | 43 (11%) | 20 (13%) | 23 (9%) | 0.38 |
| Afro-Caribbean | 2 (1%) | 1 (1%) | 1 (0%) | 1.00 |
| Other | 3 (1%) | 2 (1%) | 1 (0%) | 1.00 |
| Body mass index (kg / m^2^) | 30.3 ± 7.1 | 29.9 ± 6.1 | 30.4 ± 7.9 | 0.49 |
| Aetiology of end-stage liver disease |  |  |  |  |
| Alcohol | 101 (25%) | 36 (23%) | 65 (26%) | 0.48 |
| Hepatitis B | 3 (1%) | 1 (1%) | 2 (1%) | 1.00 |
| Hepatitis C | 52 (13%) | 21 (17%) | 31 (13%) | 0.96 |
| Non-alcoholic steatohepatitis | 71 (18%) | 29 (19%) | 42 (17%) | 0.80 |
| Primary biliary cirrhosis | 20 (5%) | 10 (6%) | 10 (4%) | 0.43 |
| Cryptogenic | 11 (3%) | 2 (1%) | 9 (4%) | 0.27 |
| Autoimmune | 6 (1%) | 2 (1%) | 4 (2%) | 1.00 |
| Alpha-1-antitrypsin deficiency | 5 (1%) | 2 (1%) | 3 (1%) | 1.00 |
| Other | 135 (33%) | 35 (29%) | 100 (41%) | 0.001 |
| MELD score | 17 ± 5 | 16 ± 5 | 18 ± 7 | 0.002 |
| Cardiac risk factors |  |  |  |  |
| Diabetes | 213 (53%) | 79 (50%) | 134 (54%) | 0.70 |
| Hypertension | 43 (11%) | 17 (11%) | 26 (11%) | 0.98 |
| Hypercholesterolemia | 104 (26%) | 42 (27%) | 62 (25%) | 0.45 |
| Current smoker | 63 (16%) | 28 (18%) | 35 (14%) | 0.56 |
| Family history of CAD | 3 (1%) | 2 (1%) | 1 (0%) | 0.52 |
| Symptomatic chest pain | 14 (3%) | 2 (1%) | 12 (5%) | 0.27 |
| History of revascularization (PCI or CABG) | 14 (3%) | 5 (3%) | 9 (4%) | 0.79 |
| History of myocardial infarction | 9 (2%) | 5 (3%) | 4 (2%) | 0.32 |
| Medications |  |  |  |  |
| Aspirin | 39 (10%) | 17 (11%) | 22 (9%) | 0.55 |
| Beta-blocker | 172 (43%) | 77 (49%) | 95 (39%) | 0.47 |
| ACE inhibitor / Angiotensin receptor blocker | 41 (10%) | 15 (9%) | 26 (11%) | 0.72 |
| Calcium channel blocker | 3 (1%) | 2 (1%) | 1 (0%) | 1.00 |
| Loop diuretic | 86 (21%) | 40 (25%) | 46 (19%) | 0.39 |
| Mineralocorticoid receptor antagonist | 128 (32%) | 50 (32%) | 78 (32%) | 0.99 |
| Statin | 86 (21%) | 35 (22%) | 51 (21%) | 0.73 |
| Insulin | 209 (52%) | 77 (49%) | 132 (54%) | 0.39 |
| Hemoglobin (g / L) | 114 (99 – 128) | 117 (105−129) | 111 (97 – 126) | 0.74 |
| Total cholesterol (mg / dL) | 152 ± 61 | 157 ± 54 | 147 ± 50 | 0.28 |
| INR | 1.42 ± 0.90 | 1.36 ± 0.33 | 1.46 ± 1.16 | 0.29 |
| Creatinine (mg / dL) | 0.97 (0.75-1.25) | 0.90 (0.75−1.15) | 1.01 (0.77 – 1.30) | 0.71 |

Data are N (%), mean ± SD or median (interquartile range).

Differences between patients who did or did not proceed to liver transplantation were analyzed using independent samples Student t, chi-square or Fisher exact tests, where appropriate.

*Patients excluded from the primary analysis included two patients who underwent early revascularization within 90 days of the SPECT MPI imaging to remove cardiovascular outcomes driven temporally by this result.


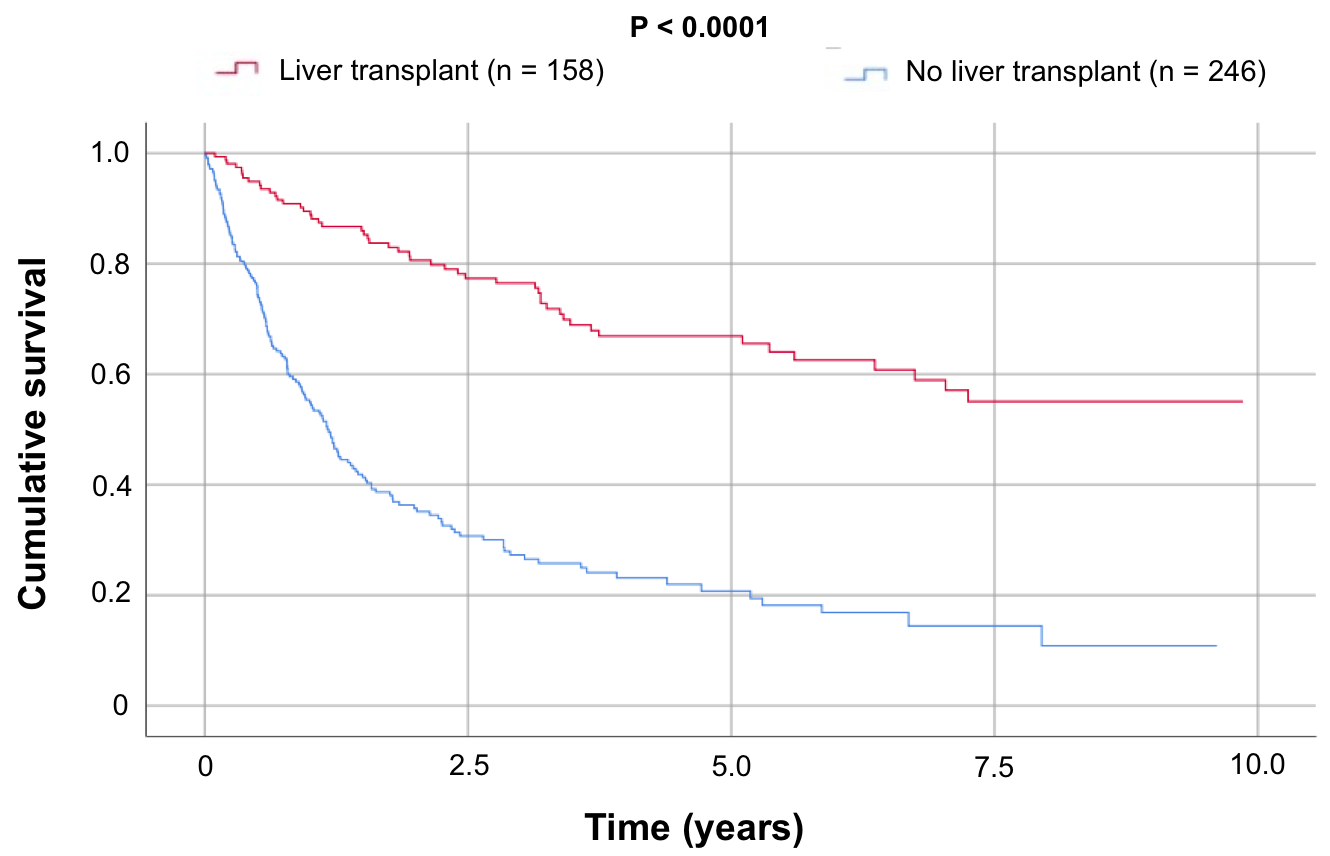


**Figure S1. Kaplan-Meier curve for unadjusted cumulative survival from**

**all-cause death dichotomized according to the occurrence of orthotopic liver transplantation.**

Two-sided generalized Wilcoxon tests were used to determine significance.

**
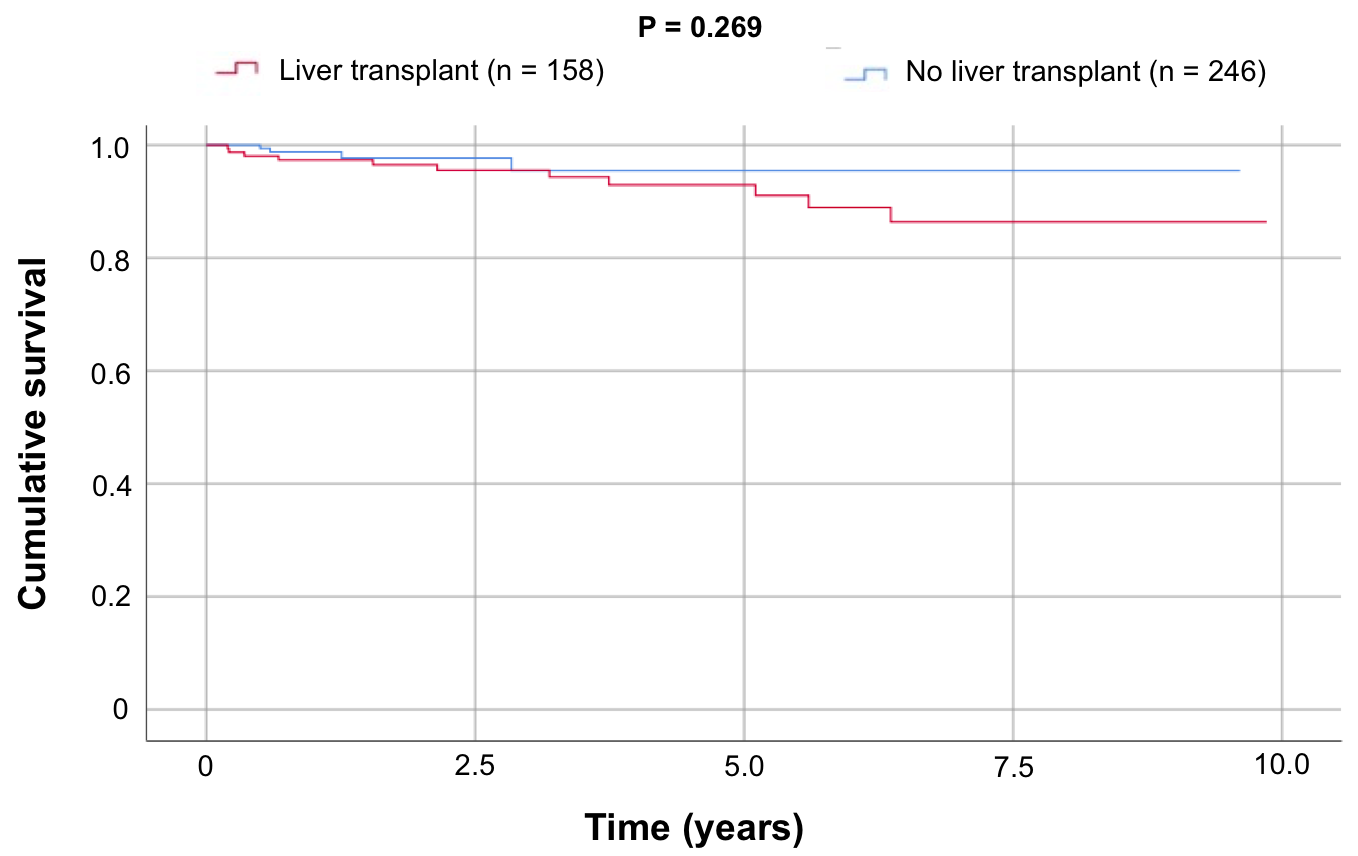
**

**Figure S2. Kaplan-Meier curve for unadjusted cumulative survival from**

**cardiovascular death dichotomized according to the occurrence of orthotopic liver transplantation.**

Two-sided generalized Wilcoxon tests were used to determine significance.
