# Supplementary material for: Prognostic value of coronary risk factors, exercise capacity and single photon emission computed tomography in liver transplantation candidates: A 5-year follow-up study
Source: J Nucl Cardiol. 2020 May 11;28(6):2876–91. doi: 10.1007/s12350-020-02126-z (PMC8709822; doi:10.1007/s12350-020-02126-z)
Supplement: Supplementary file 2 — Supplementary material 2 (PPTX 531 kb) [file 12350_2020_2126_MOESM2_ESM.pptx]

## Slide 1
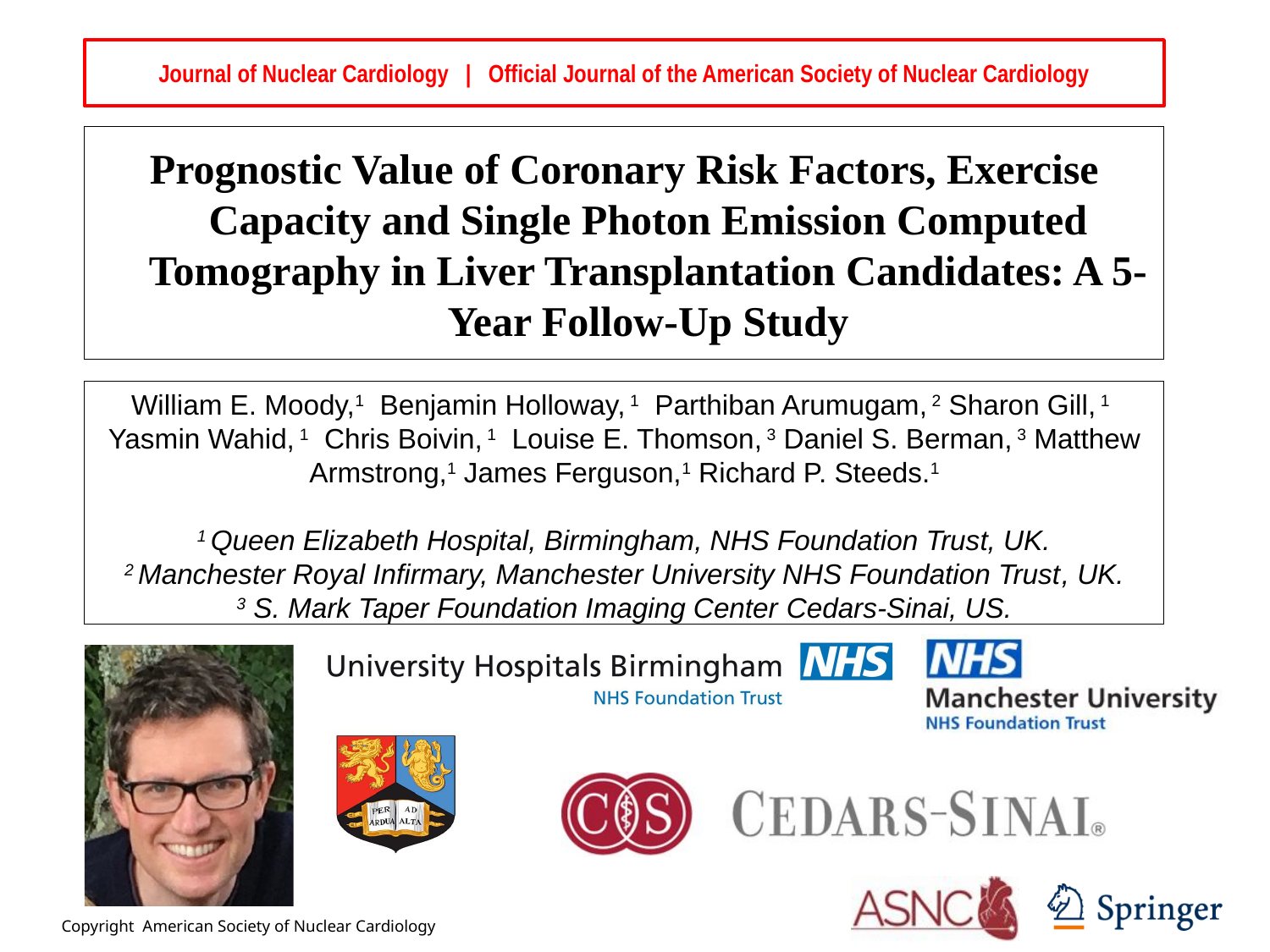

Journal of Nuclear Cardiology | Official Journal of the American Society of Nuclear Cardiology
# Prognostic Value of Coronary Risk Factors, Exercise Capacity and Single Photon Emission Computed Tomography in Liver Transplantation Candidates: A 5-Year Follow-Up Study
William E. Moody,1 Benjamin Holloway, 1 Parthiban Arumugam, 2 Sharon Gill, 1 Yasmin Wahid, 1 Chris Boivin, 1 Louise E. Thomson, 3 Daniel S. Berman, 3 Matthew Armstrong,1 James Ferguson,1 Richard P. Steeds.1
1 Queen Elizabeth Hospital, Birmingham, NHS Foundation Trust, UK.
2 Manchester Royal Infirmary, Manchester University NHS Foundation Trust, UK.
3 S. Mark Taper Foundation Imaging Center Cedars-Sinai, US.
Copyright American Society of Nuclear Cardiology

## Slide 2
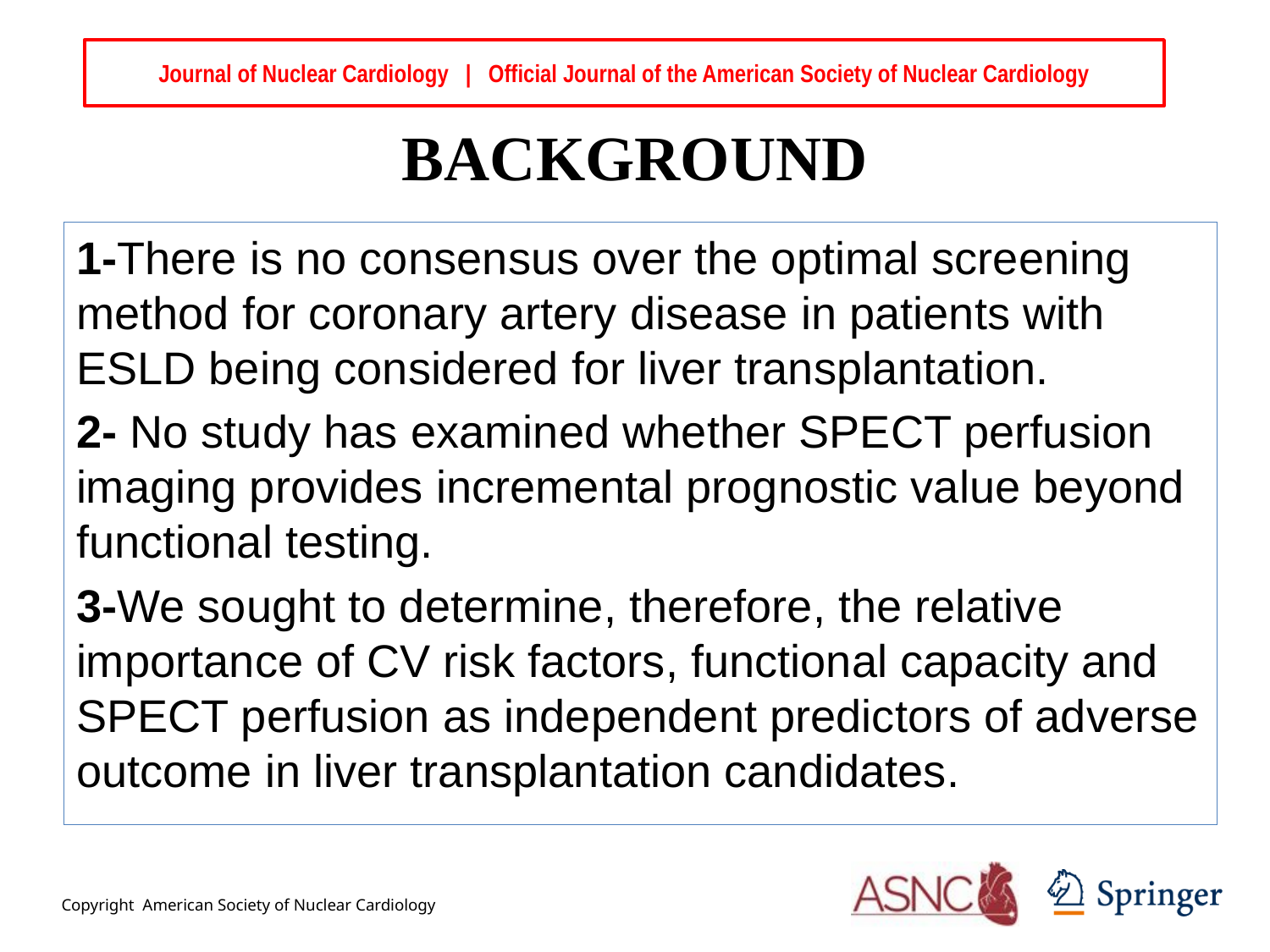

Journal of Nuclear Cardiology | Official Journal of the American Society of Nuclear Cardiology
# BACKGROUND
1-There is no consensus over the optimal screening method for coronary artery disease in patients with ESLD being considered for liver transplantation.
2- No study has examined whether SPECT perfusion imaging provides incremental prognostic value beyond functional testing.
3-We sought to determine, therefore, the relative importance of CV risk factors, functional capacity and SPECT perfusion as independent predictors of adverse outcome in liver transplantation candidates.
Copyright American Society of Nuclear Cardiology

## Slide 3
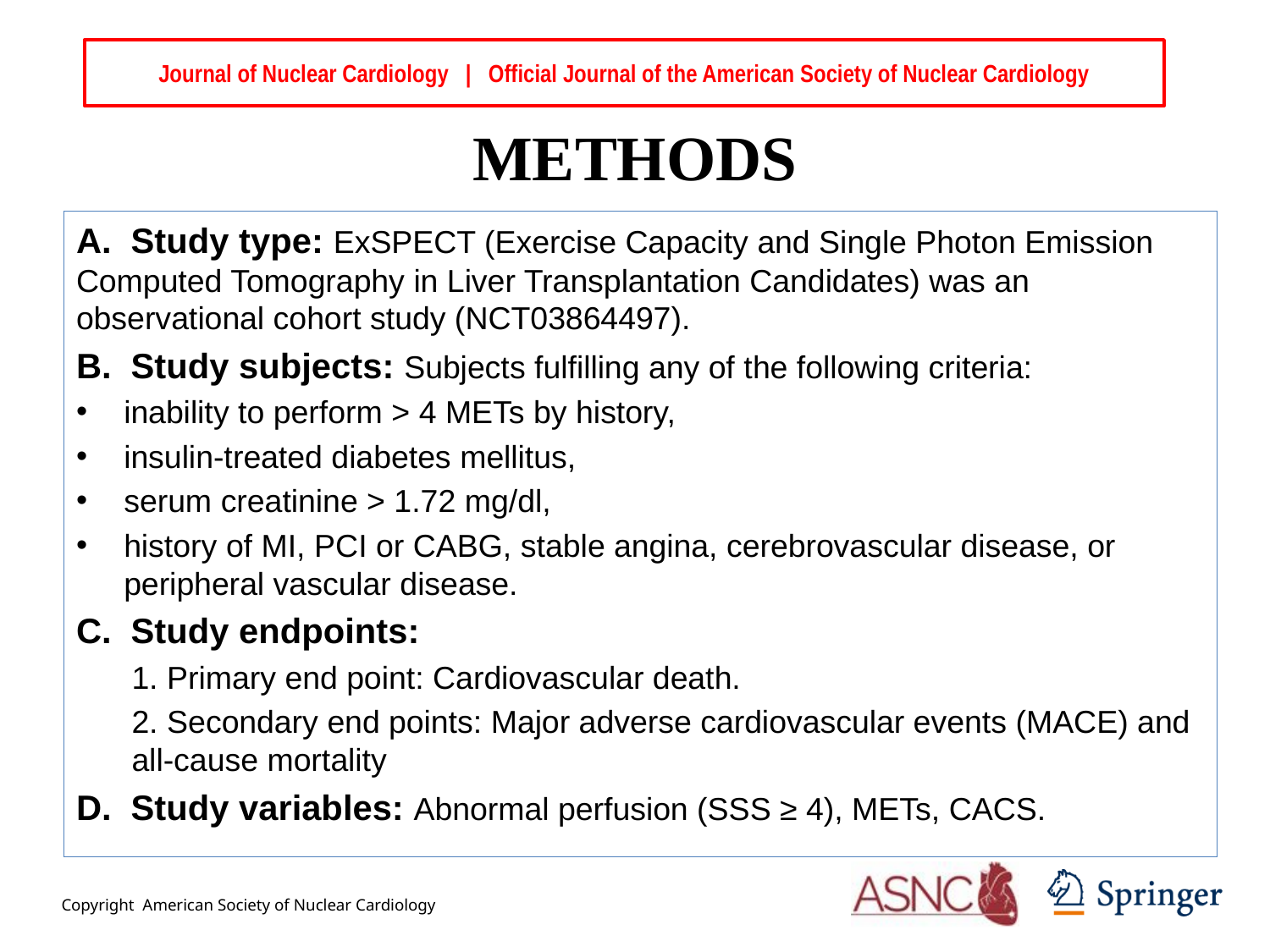

Journal of Nuclear Cardiology | Official Journal of the American Society of Nuclear Cardiology
# METHODS
A. Study type: ExSPECT (Exercise Capacity and Single Photon Emission Computed Tomography in Liver Transplantation Candidates) was an observational cohort study (NCT03864497).
B. Study subjects: Subjects fulfilling any of the following criteria:
inability to perform > 4 METs by history,
insulin-treated diabetes mellitus,
serum creatinine > 1.72 mg/dl,
history of MI, PCI or CABG, stable angina, cerebrovascular disease, or peripheral vascular disease.
C. Study endpoints:
1. Primary end point: Cardiovascular death.
2. Secondary end points: Major adverse cardiovascular events (MACE) and all-cause mortality
D. Study variables: Abnormal perfusion (SSS ≥ 4), METs, CACS.
Copyright American Society of Nuclear Cardiology

## Slide 4
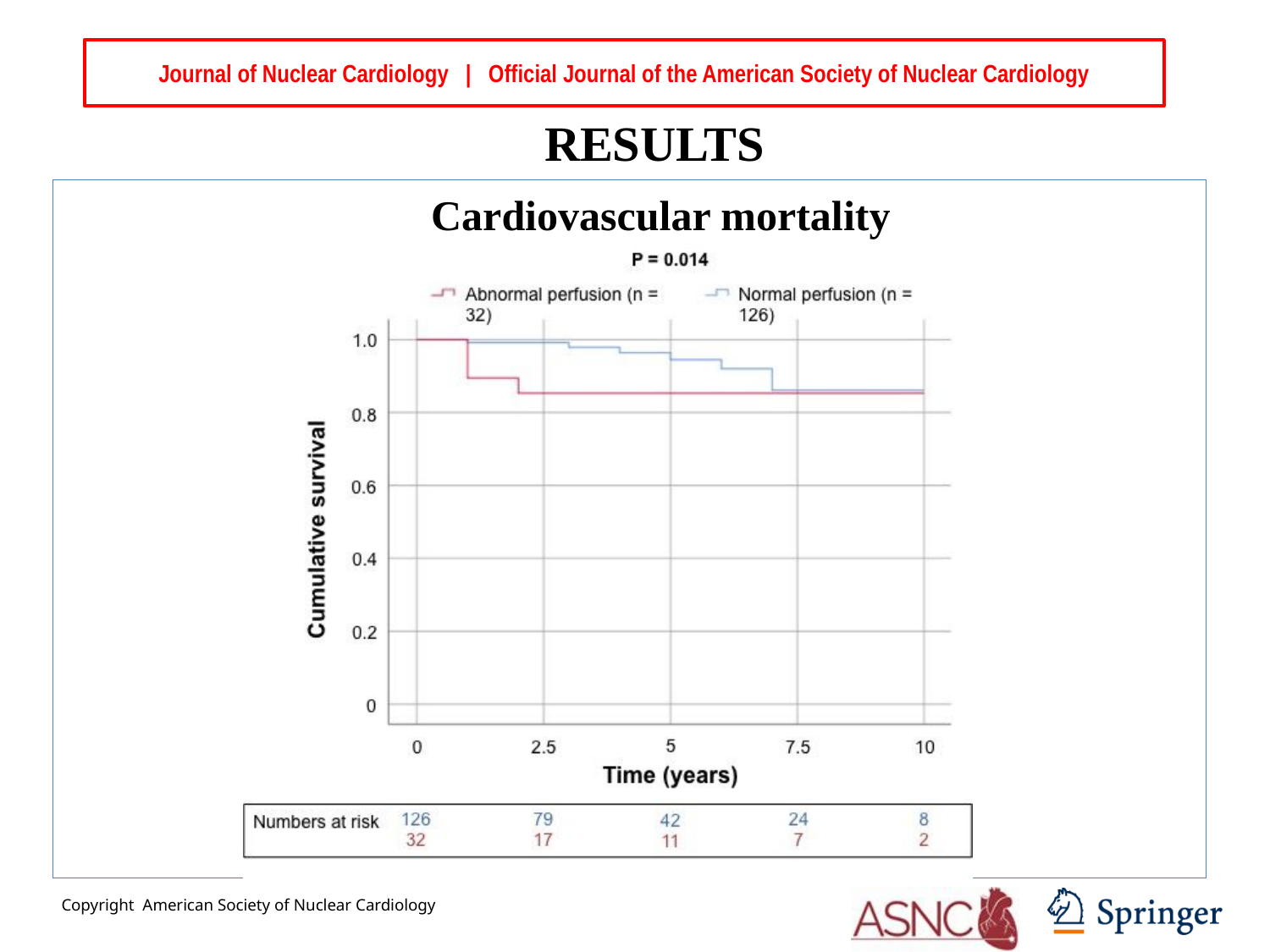

Journal of Nuclear Cardiology | Official Journal of the American Society of Nuclear Cardiology
RESULTS
Cardiovascular mortality
Copyright American Society of Nuclear Cardiology

## Slide 5
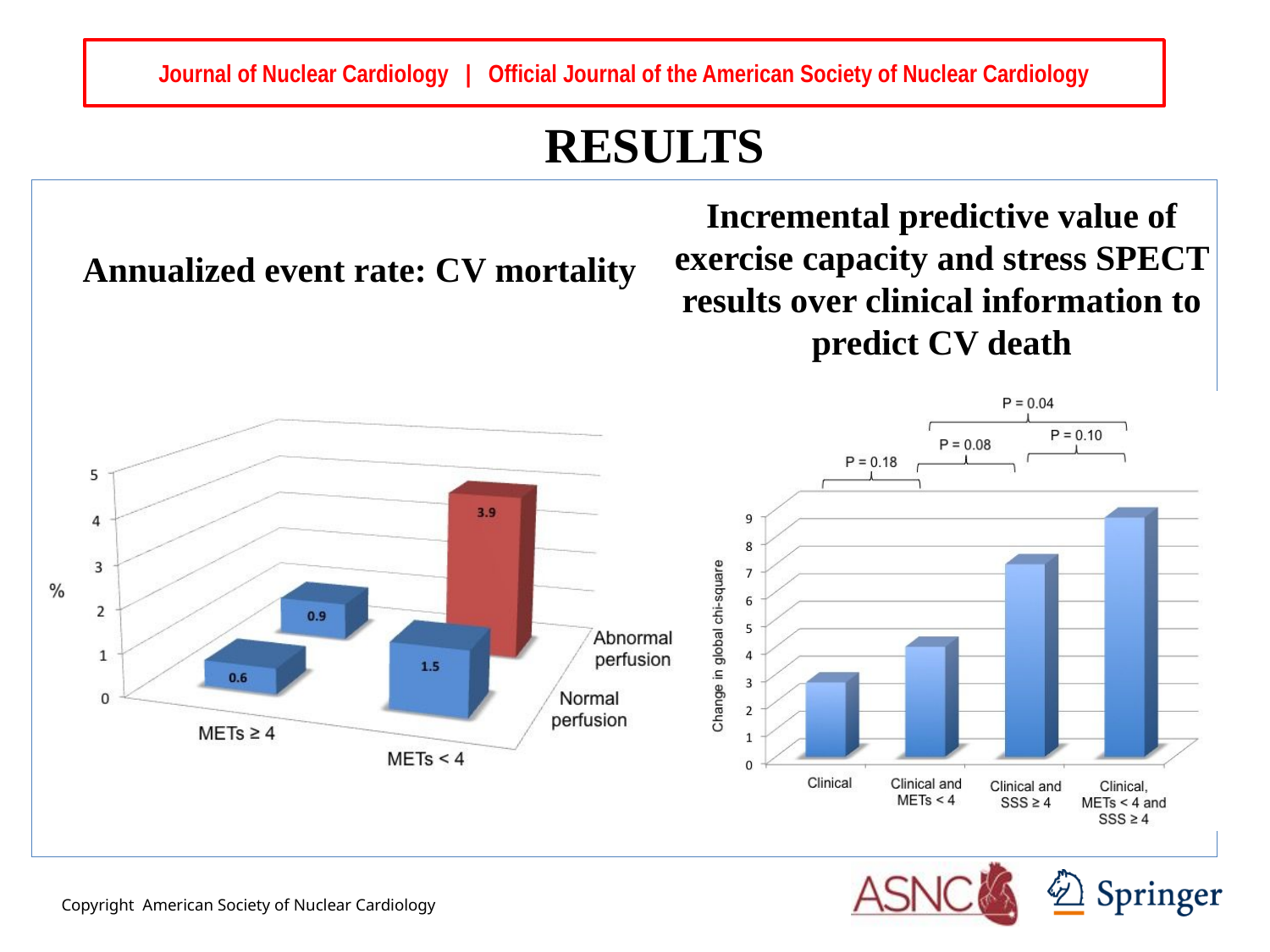

Journal of Nuclear Cardiology | Official Journal of the American Society of Nuclear Cardiology
RESULTS
Incremental predictive value of exercise capacity and stress SPECT results over clinical information to predict CV death
Annualized event rate: CV mortality
Copyright American Society of Nuclear Cardiology

## Slide 6
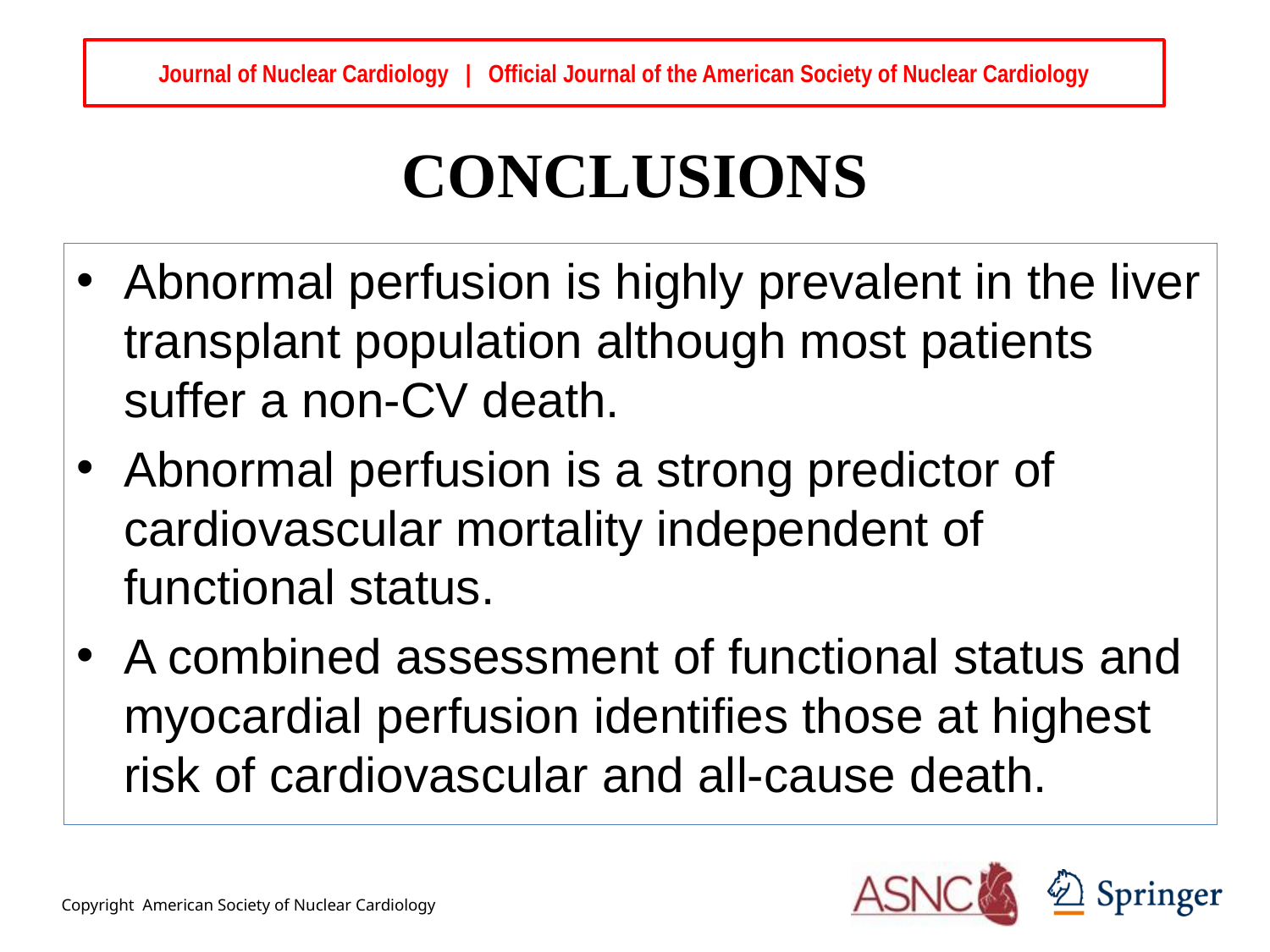

Journal of Nuclear Cardiology | Official Journal of the American Society of Nuclear Cardiology
# CONCLUSIONS
Abnormal perfusion is highly prevalent in the liver transplant population although most patients suffer a non-CV death.
Abnormal perfusion is a strong predictor of cardiovascular mortality independent of functional status.
A combined assessment of functional status and myocardial perfusion identifies those at highest risk of cardiovascular and all-cause death.
Copyright American Society of Nuclear Cardiology
